# Evidence for a role of TRIB3 in the regulation of megakaryocytopoiesis

**DOI:** 10.1038/s41598-017-07096-w

**Published:** 2017-07-27

**Authors:** Lee Butcher, Maninder Ahluwalia, Tiit Örd, Jessica Johnston, Roger H. Morris, Endre Kiss-Toth, Tõnis Örd, Jorge D. Erusalimsky

**Affiliations:** 1 School of Health Sciences, Cardiff Metropolitan University, Cardiff, UK; 2 Estonian Biocentre, Tartu, Estonia; 3 Department of Infection, Immunity and Cardiovascular Disease, University of Sheffield, Sheffield, UK

## Abstract

Megakaryocytopoiesis is a complex differentiation process driven by the hormone thrombopoietin by which haematopoietic progenitor cells give rise to megakaryocytes, the giant bone marrow cells that in turn break down to form blood platelets. The Tribbles Pseudokinase 3 gene (*TRIB3*) encodes a pleiotropic protein increasingly implicated in the regulation of cellular differentiation programmes. Previous studies have hinted that *TRIB3* could be also involved in megakaryocytopoiesis but its role in this process has so far not been investigated. Using cellular model systems of haematopoietic lineage differentiation here we demonstrate that *TRIB3* is a negative modulator of megakaryocytopoiesis. We found that in primary cultures derived from human haematopoietic progenitor cells, thrombopoietin-induced megakaryocytic differentiation led to a time and dose-dependent decrease in *TRIB3* mRNA levels. In the haematopoietic cell line UT7/mpl, silencing of *TRIB3* increased basal and thrombopoietin-stimulated megakaryocyte antigen expression, as well as basal levels of ERK1/2 phosphorylation. In primary haematopoietic cell cultures, silencing of *TRIB3* facilitated megakaryocyte differentiation. In contrast, over-expression of *TRIB3* in these cells inhibited the differentiation process. The *in-vitro* identification of *TRIB3* as a negative regulator of megakaryocytopoiesis suggests that *in-vivo* this gene could be important for the regulation of platelet production.

## Introduction

*TRIB3*, a mammalian orthologue of Drosophila *Tribbles*, is a member of an emerging group of genes encoding pseudo-kinases, which are increasingly implicated in the regulation of signal transduction and gene transcription^1, 2^. Mammals express three tribbles proteins: TRIB1, TRIB2 and TRIB3^3^. TRIB3 can bind and inhibit the protein kinases AKT^4^ and MEK1^5^, as well as interact with E3 ubiquitin ligases such as SMURF1^6^ and COP1^7^, targeting proteins for degradation. In addition, TRIB3 can act as a transcriptional repressor by interacting with a number of transcription factors, including ATF4^8^, DDIT3^9^, C/EBPβ^10^ and PPARγ^11^. Consistent with this multiplicity of binding partners and functions, *TRIB3* has been linked to the modulation of diverse biological processes, including cellular metabolism, stress responses, cell survival, proliferation and differentiation.

Recently, in a study investigating the gene expression signature of the platelet-lowering agent anagrelide we found that *TRIB3* is up-regulated when megakaryocytopoiesis is inhibited by this drug^12^. *TRIB3* has also been listed in a microarray data set among the down-regulated genes of a thrombopoietin (TPO)-response signature^13^. While these findings raise the possibility that *TRIB3* could be involved in the control of megakaryocytopoiesis, its function in this process has not been formally investigated.

Here we show that *TRIB3* expression is down-regulated during the *ex vivo* differentiation of haematopoietic progenitor cells into the megakaryocytic lineage, that silencing of this gene enhances megakaryocyte differentiation and conversely, that its over-expression inhibits this process. In addition, we provide evidence suggesting that the underlying mechanism of action of TRIB3 involves inhibition of the MAPK pathway. These findings identify *TRIB3* as a potential negative modulator of megakaryocytopoiesis in haematopoietic cells.

## Results

### *TRIB3* mRNA expression is down-regulated during megakaryocyte differentiation

**Figure 1:**
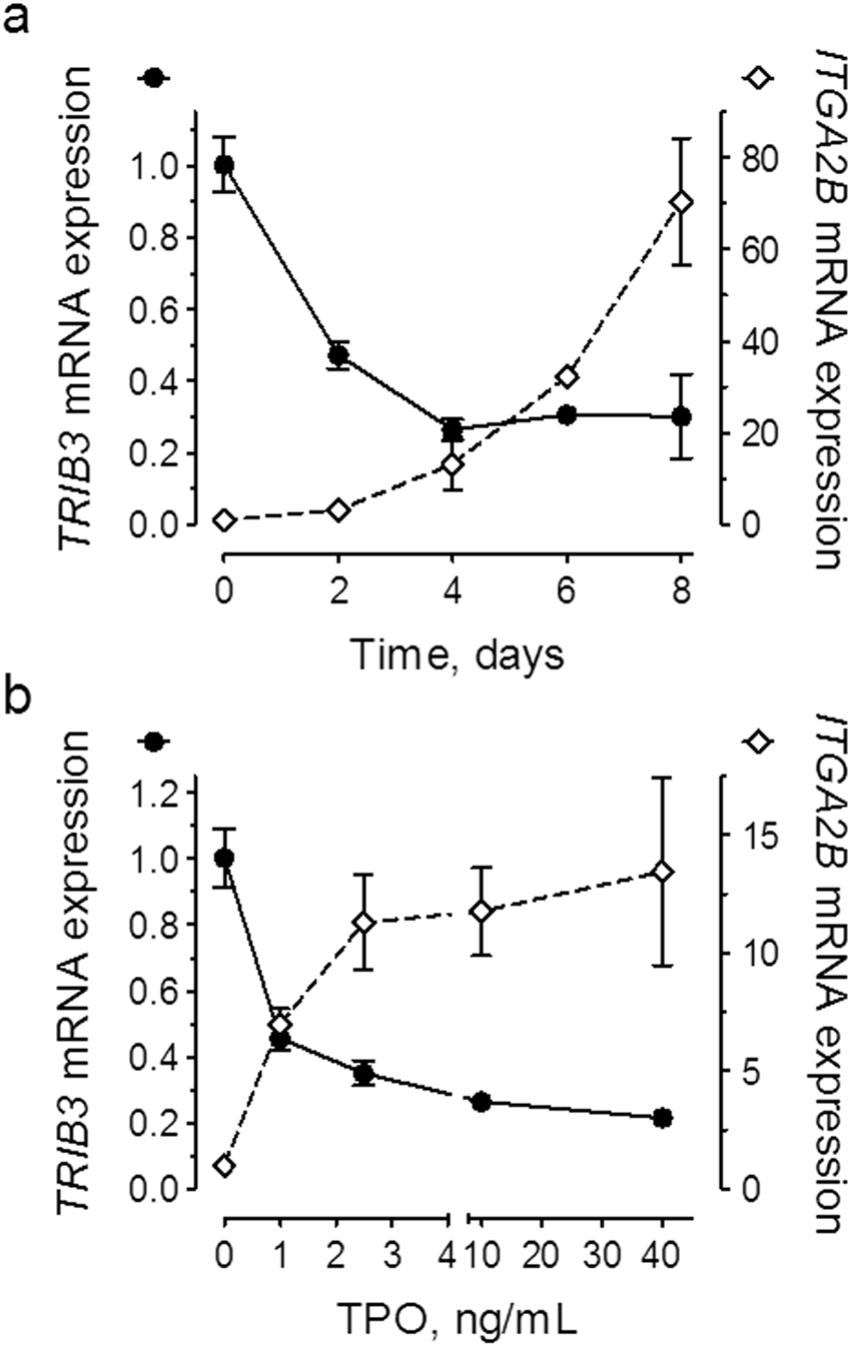
TPO induces a time and dose-dependent decrease of *TRIB3* mRNA levels. (**a**) Primary haematopoietic cells were grown under megakaryocyte differentiation conditions with 40 ng/mL TPO for the indicated lengths of time, or (**b**) for four days with the indicated doses of TPO. Gene expression levels are expressed relative to the respective transcript levels at the initiation of the culture period. Results are representative of three independent experiments. Error bars denote SD of technical replicates (n = 3–6). Error bars smaller than the size of the symbol are not shown.

To assess whether *TRIB3* could be involved in the process of megakaryocyte differentiation we initially examined its expression in cultures of primary haematopoietic cells grown in the presence of TPO. Under these conditions there was a time-dependent decrease in *TRIB3* mRNA levels, with the maximal effect (~75% reduction) being observed after four days in culture (Fig. 1a). As shown in Fig. 1b the reduction in *TRIB3* expression was maximized with increasing concentrations of TPO. Importantly, these changes were mirrored by a time- and dose-dependent increase in the expression of the megakaryocyte specific gene *ITGA2B* (aka, *GPIIb*).

### Silencing of TRIB3 expression enhances megakaryocyte differentiation

**Figure 2:**
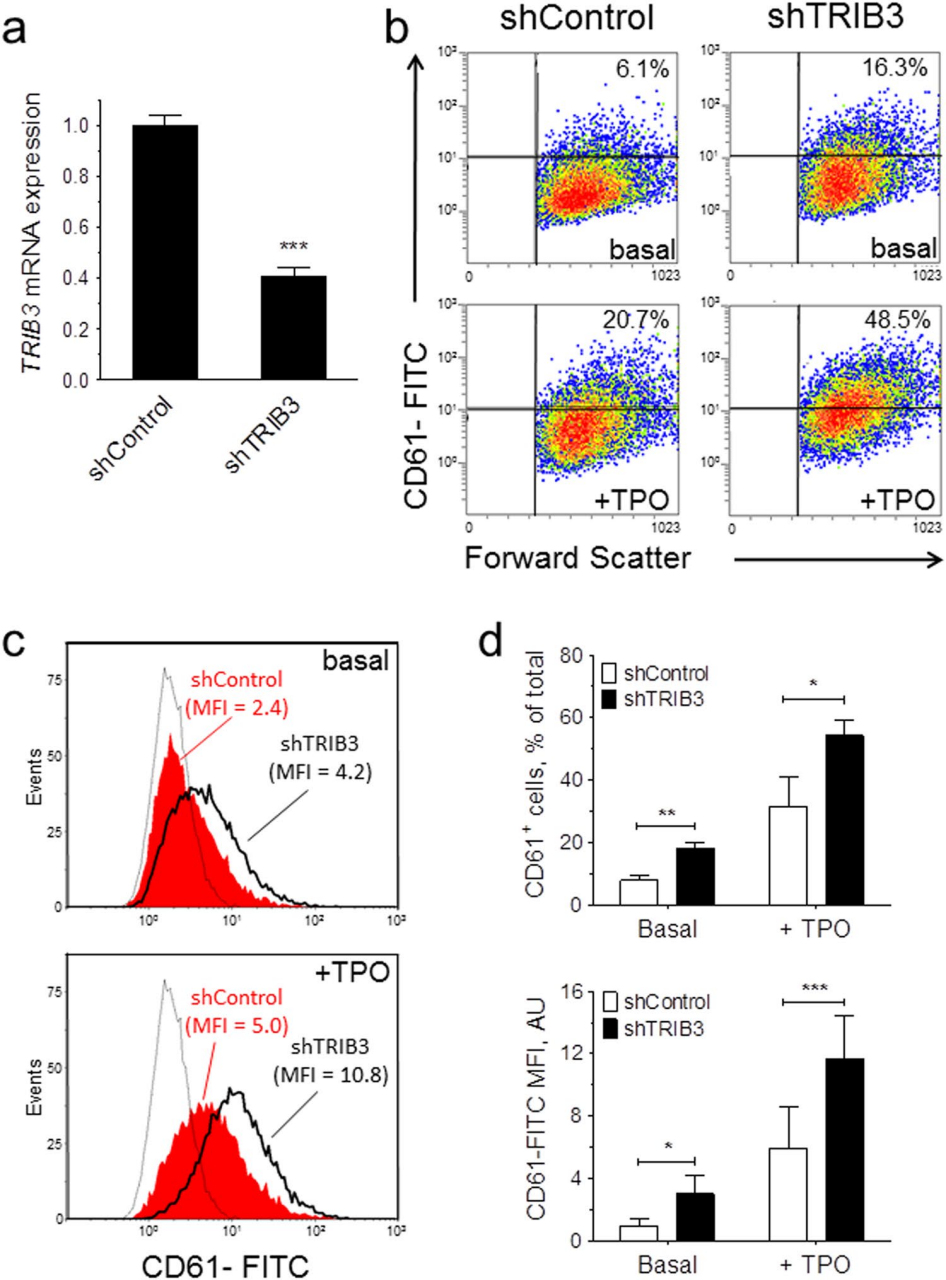
*TRIB3* silencing enhances the megakaryocytic differentiation of UT7/mpl cells. shRNA-transduced cells (shControl and shTRIB3.4) were grown for 7 days in the absence (basal) or presence of 100 ng/mL TPO (+TPO) and then analysed for *TRIB3* mRNA and CD61 expression. (**a**) *TRIB3* mRNA levels are expressed relative to the basal levels in shControl. Error bars denote SD of pooled Q-PCR data from two independent cultures (n = 6); (**b–d**) Flow cytometric analysis of megakaryocytic differentiation. (**b**) Representative density plots display the percentages of CD61^+^ cells. (**c**) Representative histogram plots display the median fluorescence intensities (MFI); the faint traces correspond to the fluorescence distribution of samples stained with an isotype-matched control antibody. (**d**) Results of three independent cultures are presented as mean ± SD. *P < 0.05, **P < 0.01, ***P < 0.001.

To test the functional significance of the above described *TRIB3* down-regulation we examined the effects of *TRIB3* silencing in the haematopoietic cell line UT7/mpl, using lentiviral-mediated shRNA interference. Five *TRIB3* shRNAs (designated shTRIB3.1 to shTRIB3.5) were initially surveyed in HT1080 cells. Of these, one was found to be ineffective (shTRIB3.1) while the other four reduced *TRIB3* expression by at least 50% (data not shown). In UT7/mpl cells three of the four positive shRNAs also caused a marked increase in the expression of the megakaryocytic surface marker CD61, under both basal and TPO-stimulated conditions, while the fourth shRNA (shTRIB3.2) only affected expression under basal conditions (Supplementary Table S1). Additional experiments demonstrated that shTRIB3.3 and shTRIB3.4 were consistently the most effective shRNAs, producing similar results. Results with shTRIB3.4 are shown in Fig. 2.

**Figure 3:**
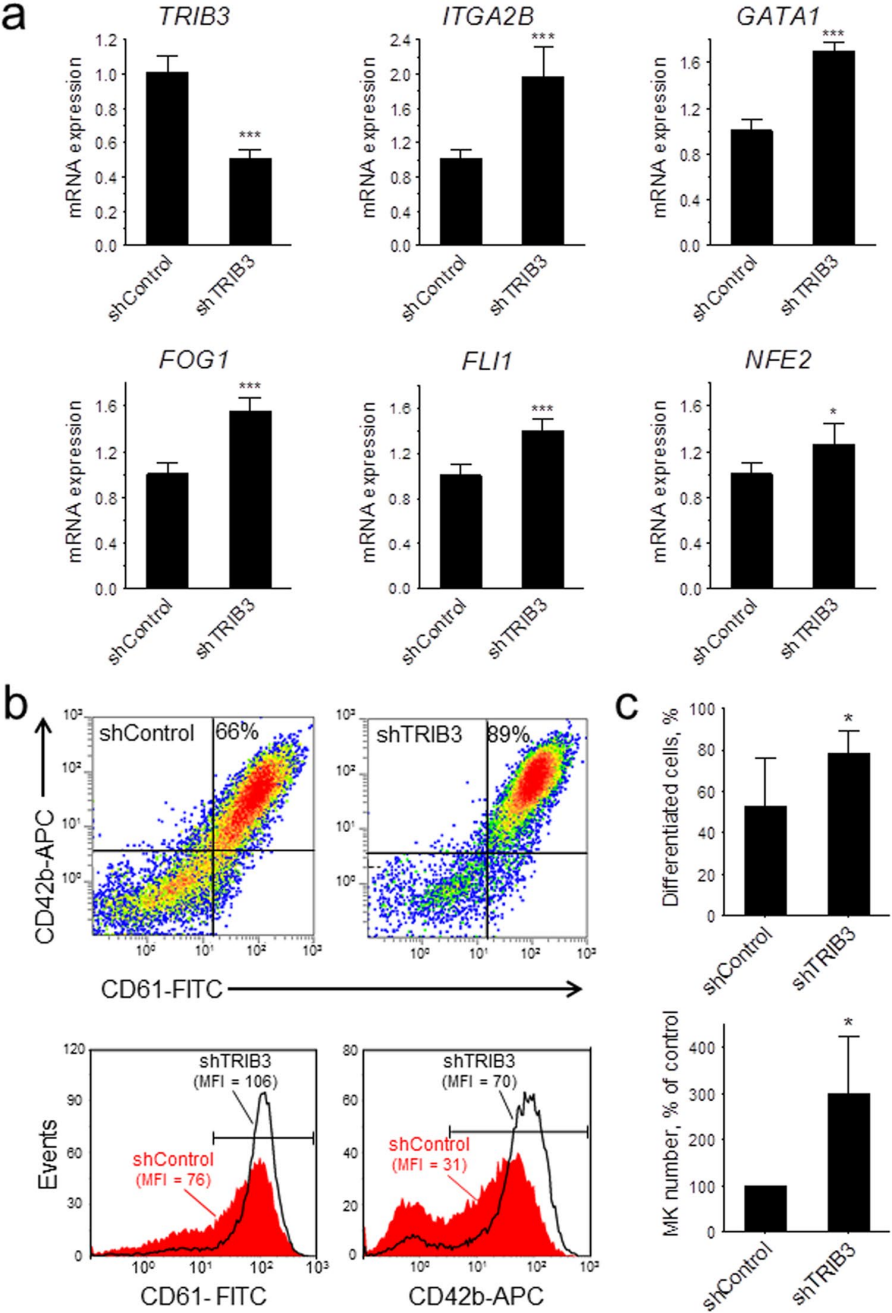
*TRIB3* silencing enhances the megakaryocytic differentiation of primary haematopoietic cells. shRNA-transduced cells (shControl and shTRIB3.4) were grown for 7 days with 40 ng/mL TPO and then analysed for mRNA and surface marker expression. (**a**) mRNA levels for the indicated genes are depicted relative to the respective levels in shControl. Error bars denote SD of pooled Q-PCR data from two independent experiments (n = 6). (**b,c**) Flow cytometric analysis of megakaryocytic differentiation. (**b**) Representative density plots display the percentages of differentiated cells and histogram plots display the median fluorescence intensities of the cells encompassed within the horizontal markers. (**c**) Results of four independent experiments are presented as mean ± SD. The top panel shows the percentages of differentiated cells in the transduced cultures and the bottom panel shows the total number of differentiated cells, expressed as a percentage of those in shControl. *P < 0.05 and ***P < 0.001.

**Figure 4:**
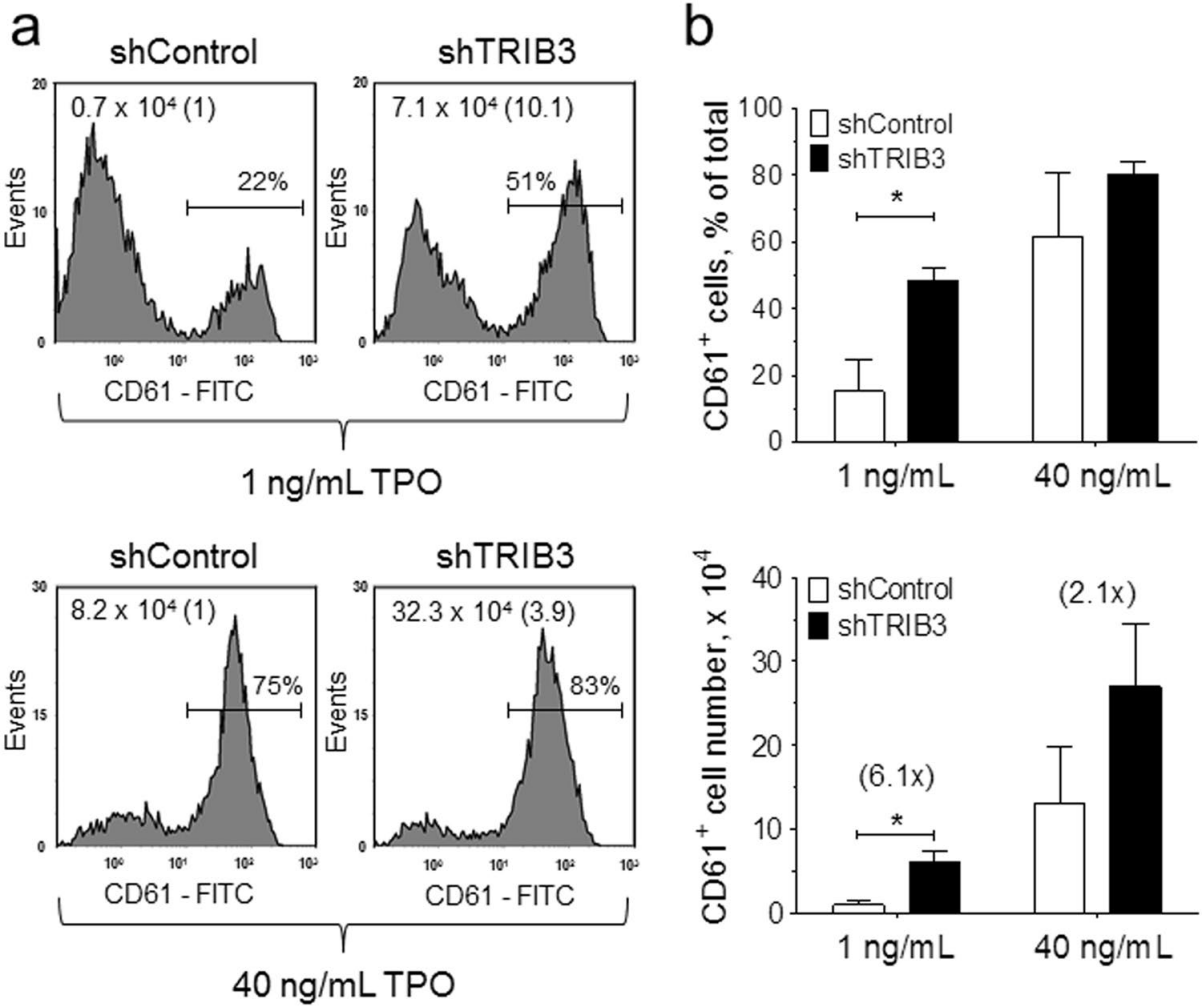
*TRIB3* silencing potentiates the megakaryocytopoietic activity of TPO. shRNA-transduced primary haematopoietic cells were grown as in Fig. 3 with either 1 or 40 ng/mL TPO and then analysed for CD61 expression. (**a**) Representative histogram plots display the percentages and total number of CD61^+^ cells in the cultures; the fold change in the number of CD61^+^ cells relative to shControl is shown in brackets. (**b**) Results of two independent experiments are presented as mean ± SD. *P < 0.05.

We next assessed whether *TRIB3* silencing affected the development of megakaryocytes in liquid cultures of CD34^+^-derived human haematopoietic cells. Initial experiments with a mixture of *TRIB3* shRNAs (shTRIB3.3, shTRIB3.4 and shTRIB3.5) showed a >50% reduction in *TRIB3* mRNA expression and a marked increase in the final number of megakaryocytes in the cultures when compared to a control shRNA (Supplementary Fig. S1). Further transductions carried out with shTRIB3.4 alone resulted in a similar degree of silencing (Fig. 3a). Furthermore, this level of silencing resulted in a >50% increase in *ITGA2B* mRNA and in similar increases in the expression of various transcription factors known to be implicated in the execution of the megakaryocyte differentiation program, namely *GATA1* ^14^, *FOG1* ^15^, *FLI1* ^16^ and *NFE2* ^17^. Accordingly, *TRIB3* silencing also resulted in noticeable increases in the surface expression of the antigens CD61 and CD42b and in the overall percentage and number of differentiated megakaryocytes in the cultures (Fig. 3b,c). The potentiating action of *TRIB3* silencing on megakaryocyte development was much more manifested at submaximal doses of TPO, both in terms of the effect on the proportion and the absolute number of CD61^+^ cells in the cultures (Fig. 4). Taken together these results indicated that *TRIB3* acts as a negative modulator of megakaryocyte differentiation.

### Over-expression of TRIB3 inhibits megakaryocyte differentiation

**Figure 5:**
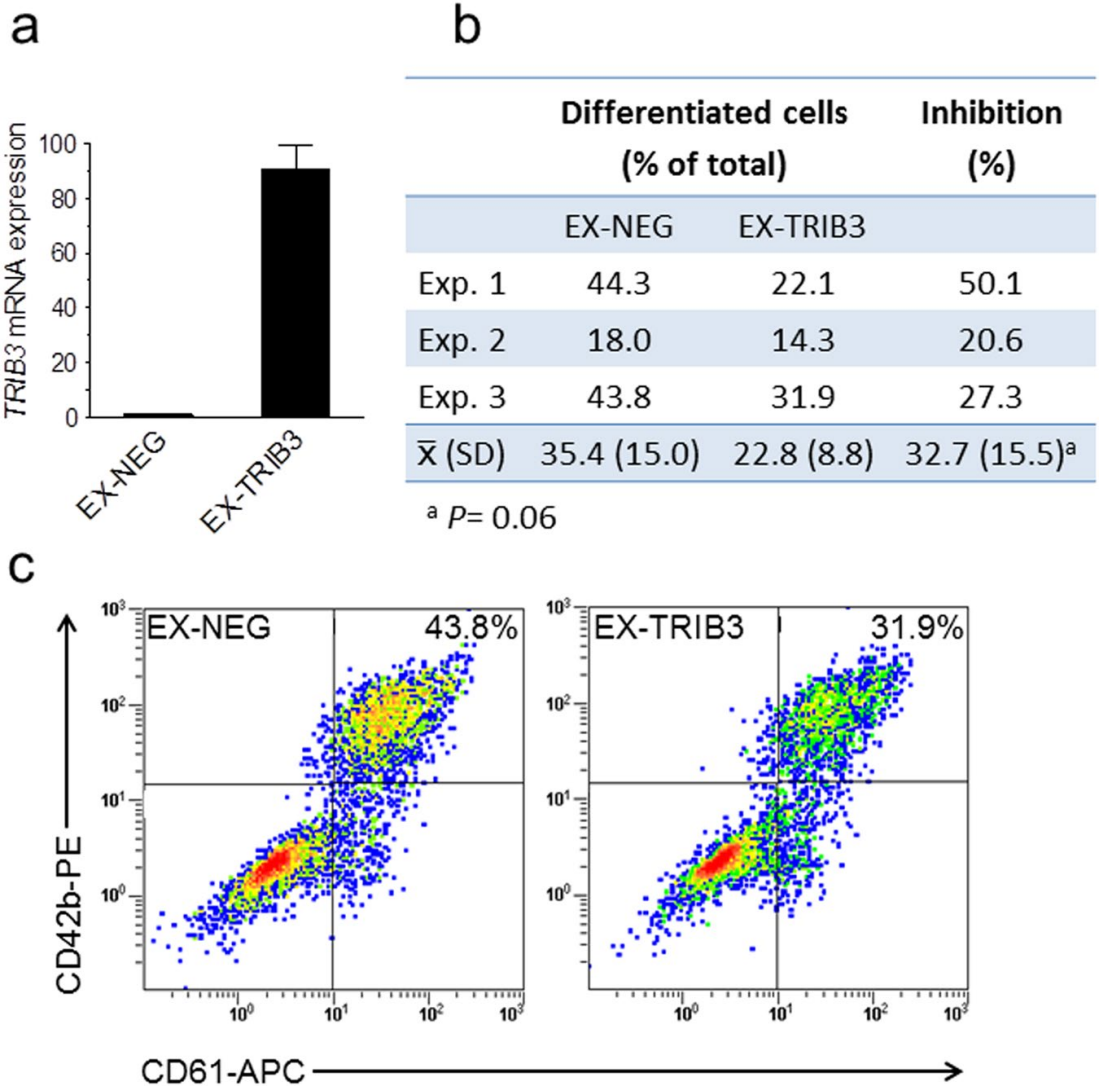
*TRIB3* over-expression inhibits the megakaryocytic differentiation of primary haematopoietic cells. Cells transduced with control (EX-NEG) or *TRIB3* (EX-TRIB3)-expressing vectors were cultured for 7 days with 1 ng/mL TPO under megakaryocyte differentiation conditions and then analysed for mRNA and surface marker expression. (**a**) *TRIB3* mRNA levels are depicted relative to the levels in EX-NEG. Error bars denote SD of technical replicates from one representative experiment (n = 6). (**b,c**) Flow cytometric analysis of differentiation. (**b**) Results of three independent experiments show the proportion of differentiated cells within the eGFP^+^ fraction; the percentage of inhibition was calculated relative to the corresponding values in EX-NEG and then averaged. (**c)** Representative density plots corresponding to Exp. 3 display the percentages of differentiated cells.

To confirm that *TRIB3* represses megakaryocyte differentiation, we ectopically over-expressed it in CD34^+^-derived human haematopoietic cells using a dual promoter lentiviral construct which includes *eGFP* as a reporter gene. We then differentiated the infected cells under standard megakaryocyte culture conditions. As shown in Fig. 5a, compared with an empty vector control, transduction with the *TRIB3*-engineered vector resulted in a large increase in the expression of this gene. Flow cytometric analysis of the eGFP-positive cells, showed that inclusion of the *TRIB3* transgene resulted in a consistent decrease in the percentage of differentiated cells (Fig. 5b,c), thus confirming its inhibitory effect on megakaryocyte development.

### Silencing of TRIB3 expression enhances the basal levels of ERK1/2 phosphorylation

**Figure 6:**
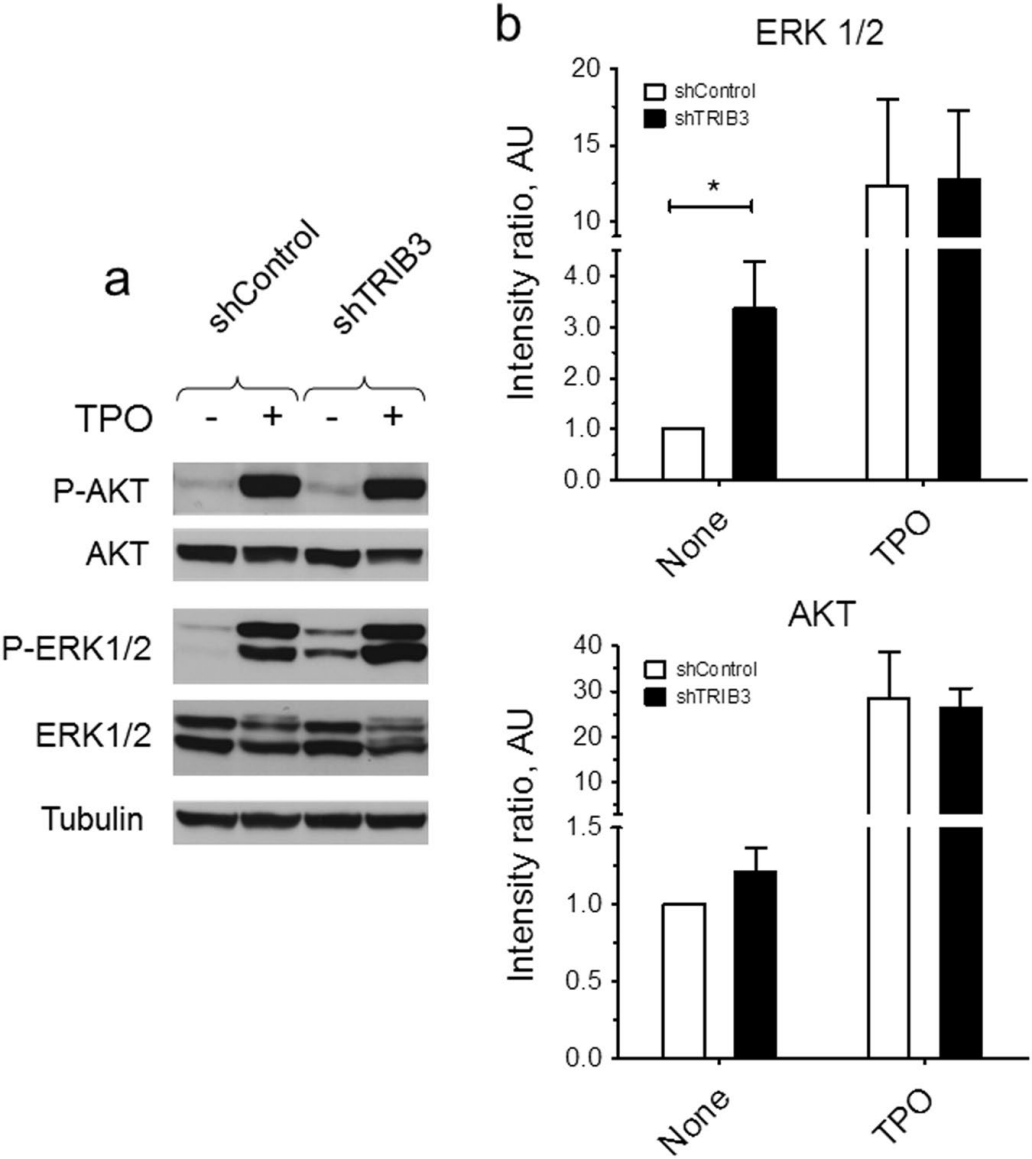
*TRIB3* silencing enhances the basal levels of ERK1/2 phosphorylation in UT7/mpl cells. shRNA-transduced cells were incubated for 30 min in the absence or presence of 100 ng/mL TPO and then analysed for AKT and ERK1/2 phosphorylation. Phosphoproteins were detected by immunoblotting and quantified by scanning densitometry. A representative immunoblot is shown in (**a**); quantification is shown in (**b**). Relative phosphorylation levels were calculated as the ratio of the intensity of the phosphorylated polypeptide bands to the intensity of the corresponding total polypeptide bands. Results are expressed relative to the level of phosphorylation measured in unstimulated shControl cells. Error bars denote SD of three independent cultures. *P < 0.05.

To investigate the potential underlying mechanism by which *TRIB3* regulates megakaryocyte differentiation we examined the effect of *TRIB3* silencing on the phosphorylation levels of AKT and ERK1/2 in UT7/mpl cells. As shown in Fig. 6, compared to shControl-transduced cells, shTRIB3-transduced cells showed significantly higher basal levels of ERK1/2 phosphorylation. In contrast, on average, no significant differences could be detected in the basal levels of AKT phosphorylation or in the phosphorylation levels of each of these proteins following stimulation with maximal concentrations of TPO.

## Discussion

The present study strongly suggests that *TRIB3* may function as a negative modulator of megakaryocytopoiesis. This conclusion is based on the following findings: Firstly, *TRIB3* expression decreased during TPO-induced differentiation of primary haematopoietic progenitors. Secondly, silencing of *TRIB3* enhanced megakaryocyte differentiation, both in a megakaryocytic cell line and in normal haematopoietic progenitors. Thirdly, ectopic over-expression of *TRIB3* attenuated the extent of this differentiation. *TRIB3* has been previously implicated in the regulation of intracellular mechanisms that control adipocyte, muscle cell, and embryonic stem cell differentiation^10, 11, 18, 19^. Thus, our findings add to an incipient body of evidence indicating that a major function of *TRIB3* is to act as negative regulator of terminal differentiation programmes.

A salient finding of the present study is that TPO down-regulated the expression of *TRIB3* in a dose-dependent manner, with the decline mirroring the increase in the expression of *ITGA2B*. This result indicates that the down-regulation of *TRIB3* is intimately coupled to the differentiation process. In this respect, it was also notable that the potentiating effect of *TRIB3* silencing on the extent of differentiation was more manifested at sub-maximal concentrations of TPO, suggesting that the presence of TRIB3 interferes with the action of the hormone and hence, that its down-regulation is required for the full effect of TPO to be manifested. TPO drives megakaryocytopoiesis by binding to the MPL receptor and thereby choreographing changes in the activity, deployment and/or expression of a complex network of signalling proteins and transcription factors, which ultimately coordinate a specific programme of gene expression^20, 21^. Precisely how TRIB3 interacts with this network is presently unknown. However, a plausible scenario is that it normally represses a pathway that transduces a MPL differentiation signal, and that one of the primary actions of TPO is to down-regulate *TRIB3* expression to de-repress this pathway. This possibility is entirely consistent with the notions that firstly, TRIB3 is known to inhibit the protein kinase MEK1^5^, which is also a down-stream effector of MPL, secondly, that strong, long-lasting activation of the MAPK pathway is required for TPO-induced megakaryocytic lineage commitment and differentiation^22, 23^ and thirdly, that MEK inhibitors restrict the development of megakaryocytes from human haematopoietic progenitors, giving rise to a more immature phenotype^24^. In support of this mechanism of action we found that *TRIB3* silencing enhanced the basal phosphorylation levels of ERK1 and ERK2, which are downstream MEK1 substrates. Importantly, while TPO also signals through AKT and despite the fact that in other systems AKT is susceptible to regulation by TRIB3^4, 18^, in UT7/mpl cells the effect of *TRIB3* silencing appeared to be circumscribed specifically to the MAPK pathway. Interestingly, *TRIB3* silencing did not affect ERK1/2 phosphorylation when cells were stimulated with maximal concentrations of TPO, seemingly suggesting that strong MPL signalling may overcome the inhibition of MEK1 by TRIB3. Thus, more detailed phosphorylation studies with submaximal concentrations of TPO will be required to determine the extent to which TRIB3 affects TPO signalling through this pathway. Furthermore, while these findings suggest that mechanistically *TRIB3* fulfils a specific role in the regulation of megakaryocytopoiesis, namely the inhibition of MEK1, they do not rule out the possibility that additionally it interacts directly with the transcription machinery that controls differentiation, as it was shown to be the case in cellular models of adipogenesis^10, 11^.

In the present work we found that silencing of *TRIB3* enhanced the TPO-stimulated increase in the mRNA levels of *GATA1*, *FOG1, FLI1* and *NFE2*. *GATA1* and *FOG1* are essential for complete development of the megakaryocytic lineage, being required from the progenitor stage throughout to the final stages of differentiation^14, 15^ whereas *FLI1* and *NFE2* act at late stages, affecting primarily the early maturing megakaryocyte^16, 25^ and pro-platelet formation^17^, respectively. Thus, while these results clearly support the conclusion that TRIB3 suppresses megakaryocytopoiesis, they do not provide an answer as to which specific stage(s) of the process it inhibits. In this context it is noteworthy that a recent study has implicated TRIB3 as a positive modulator of stress erythropoiesis^26^. Given that the differentiation of the erythroid and megakaryocytic lineages are intimately ligated, sharing common progenitors and overlapping signal transduction pathways, our results are also compatible with the possibility that *TRIB3* could be involved in the regulation of lineage fate decisions at the megakaryocytic/erythroid bi-potential progenitor stage. It would be therefore also interesting to analyse if *Trib3* deficiency affects the balance between erythropoiesis and megakaryocytopoiesis under conditions of experimental-induced anaemia.

The dysfunction of genes known to regulate megakaryocytopoiesis has been found in some cases to cause platelet disorders^21, 27^. Whether *TRIB3* also falls into this category remains to be established. In this respect, the present findings, together with our previous work showing that a major transcriptional effect of the platelet-lowering agent anagrelide was to increase *TRIB3* expression^12^, highlight the potential role that this gene could play, both as a biomarker and as drug target in the development of approaches to modulate platelet counts in diverse pathological states.

## Methods

### UT7/mpl cell culture

UT7/mpl cells (clone 5.1)^28^ were propagated in Iscove’s modified Dulbecco’s medium (GIBCO Life Technologies, UK) supplemented with 10% foetal calf serum (Hyclone, Perbio Sciences, UK), 2.5 ng/mL granulocyte-macrophage colony-stimulating factor (R&D Systems) and 0.5 mg/mL G418 as previously described^29^. To induce megakaryocytic differentiation cells were subcultured in fresh medium containing 100 ng/mL TPO. Cultures were maintained at 37 °C in a humidified incubator under 5% CO_2_/ 95% air.

### Primary megakaryocyte culture

Megakaryocytes were generated *ex-vivo* from human umbilical cord blood-derived CD34^+^ cells in a two-step liquid culture system, as previously described^12^. Briefly cryopreserved CD34^+^ cells (Stem Cell Technologies, Vancouver, Canada) were first thawed and expanded for 4–5 days in Stemspan^TM^ medium (Stem Cell Technologies) supplemented with 2% human umbilical cord blood plasma and a mixture of haematopoietic growth factors consisting of TPO (Insight, UK), SCF, Flt3 ligand and IL-3 (all from R&D Systems, Abingdon, UK). Subsequently, to promote terminal differentiation cells were sub-cultured for up to ten days in fresh medium supplemented with 2% cord blood plasma and TPO only, at the indicated concentrations.

### Lentiviral particles and cell transductions

TRC1-pLKO.1-puro plasmids carrying short hairpin sequences targetted against *TRIB3* (shTRIB3) or a non-targeting control sequence (shControl) were obtained from the Mission TRC shRNA library (Sigma-Aldrich, Dorset, UK). Short hairpin sequences are listed online in the Supplementary Table S2. The lentiviral expression constructs EX-V1552-Lv201, which contains the protein-coding open reading frame of *TRIB3*, and the negative control EX-NEG-Lv201 were purchased from Genecopoeia (Rockville, MD, USA). The packaging plasmid psPAX2 and the envelop plasmid pMD2.G were obtained from AddGene (Cambridge, MA, USA). Lentiviral particles were produced by co-transfection of HEK293T cells. Pseudoviruses were harvested after 48 h and concentrated by ultrafiltration on Amicon Ultra-15 100K filters (Millipore UK, Hertforshire, UK). Lentiviral titers were determined by transduction of HT1080 cells followed by drug selection and counting of puromycin-resistant colonies (for shRNAs) or by flow cytometric determination of eGFP^+^ cells (for lentiviral expression constructs).

Infection of UT7/mpl cells with lentiviral shRNAs was carried out by spinoculation at 900 g for 30 min at 21 °C in the presence of 4 μg/mL polybrene (Sigma-Aldrich, Dorset, UK) and at a MOI of 5, followed by overnight culture. Transduced cells were then selected in the presence of 1 μg/mL puromycin (Invitrogen, Paysley, UK).

Infection of CD34^+^ cells with lentiviral shRNAs was carried out after one day of expansion by spinoculation at 1450 g for 90 min at 21 °C in the presence of 4 μg/mL polybrene and at a MOI of 8, followed by overnight culture. shRNA-transduced cells were then selected with 1 μg/mL puromycin for two days and then grown for a further 24 h in fresh normal expansion medium prior to culture under differentiation conditions.

Infection of CD34^+^ cells with lentiviral expression vectors was carried out after 4 days of expansion by two consecutive spinoculations of 90 min each at 1450 g and 21 °C, separated by a 4 h interval, in the presence of 4 μg/mL polybrene and at a MOI of 10, followed by overnight culture. Expression vector-transduced cells were either differentiated without prior selection (for flow cytometric analysis) or were selected with puromycin for the last two days of differentiation (for mRNA analysis).

### Analysis of megakaryocytic lineage differentiation

Megakaryocytic differentiation was monitored by flow cytometry as previously described^30^ using fluorescein isothiocyanate (FITC)-, R-phycoerythrin (RPE)- or allophycocyanin (APC)-conjugated monoclonal antibodies against CD61 (clone Y2/51, Dako, Ely, UK) and/or CD42b (clone HIP1, BD Biosciences, Oxford, UK). Dead cells were excluded using 7-amino actinomycin D (1 μg/mL, Invitrogen, Paisley, UK) or near-IR fluorescent reactive dye (1:250 dilution, Invitrogen, Paisley, UK), as appropriate. In experiments where the relative antigen expression was estimated using the median fluorescent intensity, nonspecific fluorescence of cells stained with an isotype-matched control antibody was subtracted from all determinations. When indicated, the number of differentiated cells was estimated by multiplying the percentage of antigen-positive cells by the total number of live cells in the culture.

### RNA expression

Cellular RNA was extracted and then analysed by the quantitative polymerase chain reaction (Q-PCR) using gene-specific TaqMan probes (Applied Biosystems) as previously described^31^. Relative mRNA expression levels were calculated by the comparative cycle threshold (*C*_T_) method using β-glucuronidase (*GUSB*) or TATA-box binding protein (*TBP*) as internal references. Probes are listed online in the Supplementary Table S3.

### Protein phosphorylation

AKT and ERK1/2 phosphorylation was examined by immunoblotting as previously described^31^. Primary antibodies are listed online in the Supplementary Table S4.

### Statistical analysis

Differences between means were compared by a two tailed Student’s *t-*test (Graph Pad Prism, release 5.0). A value of p < 0.05 was considered to denote statistical significance.

## Electronic supplementary material

Supplementary Information
