# Supplementary material for: Evidence for a role of TRIB3 in the regulation of megakaryocytopoiesis
Source: Sci Rep. 2017 Jul 27;7:6684. doi: 10.1038/s41598-017-07096-w (PMC5532315; doi:10.1038/s41598-017-07096-w)
Supplement: Supplementary file 1 — Supplementary Information [file 41598_2017_7096_MOESM1_ESM.pdf]

Evidence for a role of TRIB3 in the regulation of megakaryocytopoiesis

Lee Butcher[1], Maninder Ahluwalia[1], Tiit Örd[2], Jessica Johnston[3], Roger H. Morris[1], Endre Kiss-Toth[3], Tõnis Örd[2], and Jorge D. Erusalimsky[1]

[1] School of Health Sciences, Cardiff Metropolitan University, Cardiff, UK;

[2] Estonian Biocentre, Tartu, Estonia;

[3] Department of Infection, Immunity and Cardiovascular Disease, University of Sheffield, Sheffield, UK

Supplementary Tables and Figure

Table S1: CD61 expression in UT7/mpl cells transduced with different *TRIB3* shRNAs

	Relative *TRIB3* mRNA expression [b]	CD61 expression (basal)		CD61 expression (+ TPO)	
		Normalized MFI, AU [c]	$CD61^{+}$ %	Normalized MFI, AU [c]	$CD61^{+}$ %
**EXPERIMENT I** [a]					
**shControl**	1.00±0.10	1.0 (0.8)	9.4	1.0 (6.1)	37.1
**shTRIB3.3**	0.33±0.04	1.9	17.5	1.6	51.3
**shTRIB3.4**	0.42±0.03	3.0	20.1	1.9	57.7
**EXPERIMENT II** [a]					
**shControl**	1.01±0.11	1 (0.8)	3.5	1 (5.8)	30.9
**shTRIB3.2**	0.47±0.08	1.5	7.0	0.8	29.1
**shTRIB3.5**	0.38±0.05	2.7	13.3	1.4	43.0

[a] shRNA-transduced cells were grown for 6 days in two separate experiments, in the absence (basal) or presence of 100 ng/mL TPO (+TPO) and then analysed for *TRIB3* mRNA and CD61 expression.

[b] Basal *TRIB3* mRNA levels are expressed relative to shControl. Values represent the mean and SD of technical replicates (n=6).

[c] The median fluorescence intensity (MFI) was normalised to the values (shown between brackets) displayed by shControl-infected cells analysed in parallel.

Table S2. List of short hairpin sequences

Plasmid	Short hairpin sequence
shControl	CCGGCAACAAGATGAAGAGCACCAACTCGAGTTGGTGCTCT TCATCTTGTTGTTTTT
shTRIB3.1	CCGGCCAGGTCCATACTCTAGGTTTCTCGAGAAACCTAGAGT ATGGACCTGGTTTTTG
shTRIB3.2	CCGGTGGATGACAACTTAGATACCGCTCGAGCGGTATCTAA GTTGTCATCCATTTTTG
shTRIB3.3	CCGGGCTAGTTCTTGTCTAACTCAACTCGAGTTGAGTTAGAC AAGAACTAGCTTTTTTG
shTRIB3.4	CCGGGATCTCAAGCTGTGTCGCTTTCTCGAGAAAGCGACAC AGCTTGAGATCTTTTTTG
shTRIB3.5	CCGGCCCAACCCGATCCCATCTCTGCTCGAGCAGAGATGGG ATCGGGTTGGGTTTTTTG

Table S3. List of gene-specific TaqMan probes

Gene symbol	Probe ID
FLI1	Hs00231107_m1
FOG1	Hs00419119_m1
GATA1	Hs00231112_m1
GUSB	Hs99999908_m1
ITGA2B	Hs00166246_m1
NFE2	Hs00232351_m1
TBP	Hs99999910_m1
TRIB3	Hs00221754_m1

Table S4. List of primary antibodies

Antigen	Source[a]; dilution
Phospho-AKT (Ser 473)	Rabbit polyclonal; 1:2000
AKT (pan)	Rabbit polyclonal; 1:4000
Phospho-p44/42 MAPK (Thr202/Tyr204)	Rabbit polyclonal; 1:4000
p44/42 MAPK (pan)	Rabbit IgG (clone 137F5); 1:4000
Alpha-tubulin	Rabbit polyclonal; 1:4000

[a] Antibodies were sourced from Cell Signaling Technologies, MA, USA.

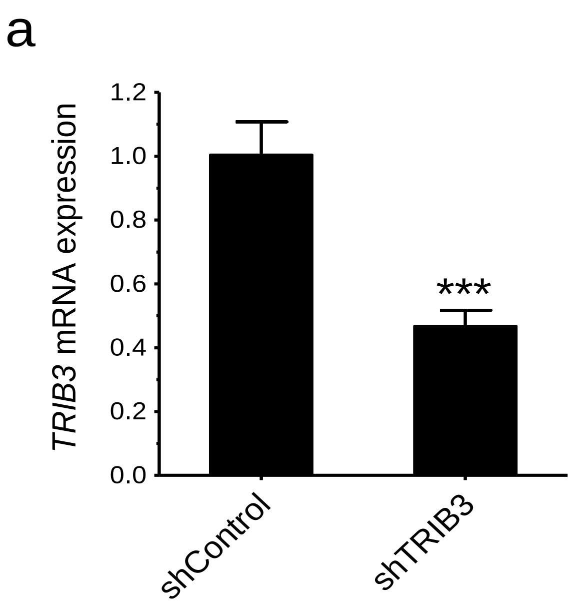

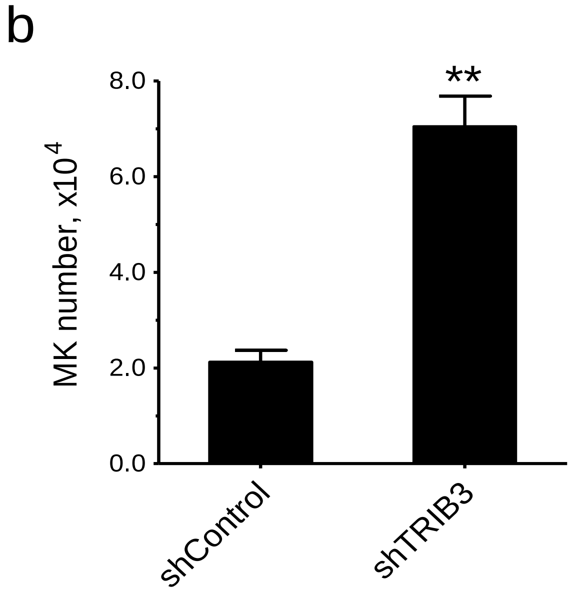

Figure S1. *TRIB3* silencing enhances megakaryocyte growth. Primary hematopoietic cells were transduced with a mixture of *TRIB3* shRNAs (TRIB3.3, TRIB3.4 and TRIB3.5) and then grown under megakaryocyte differentiation conditions. (**a**) *TRIB3* mRNA levels are expressed relative to the values in shControl (n=6). (**b**) Total number of differentiated cells in the cultures (n=2). Error bars denote SD of pooled data from two experiments; ** P<0.01 and *** P<0.001.
